# Use of pulmonary CT angiography with low tube voltage and low-iodine-concentration contrast agent to diagnose pulmonary embolism

**DOI:** 10.1038/s41598-017-13077-w

**Published:** 2017-10-16

**Authors:** Xuemei Hu, Liya Ma, Jinhua Zhang, Zhen Li, Yaqi Shen, Daoyu Hu

**Affiliations:** Department of Radiology, Tongji Hospital Affiliated to Tongji Medical College of Huazhong University of Science and Technology, Wuhan, China

## Abstract

Pulmonary CT angiography (CTPA) is regarded as the preferred imaging method in diagnosing pulmonary embolism (PE). Considering the harm of radiation exposure and the side effect of iodinated contrast agent, CTPA protocol with low tube voltage and low dose of contrast agent became research hotspot in last decade. The present study evaluates the image quality, radiation dose, positive rate of PE and the location of PE with a CTPA protocol using low tube voltage (80 kVp) and low-iodine-concentration contrast agent (270 mg I/ml) in patients suspected of PE compared to a conventional CTPA protocol (120 kVp, 350 mg I/ml). The results showed that 80 kVp CTPA protocol with 40 ml 270 mg I/ml achieved equally subjective image quality and a positive rate for diagnosing PE, though the quantitative image quality was reduced compared to the 120 kVp CTPA protocol with 40 ml 350 mg I/ml administered, with a 63.6% decrease in radiation dose and a 22.9% reduction in iodine content of contrast agent. Our results document that CTPA protocol with low tube voltage and low iodine concentration of contrast agent is satisfied to the clinical application.

## Introduction

Pulmonary embolism (PE) is a common disorder that is accompanied by high morbidity (estimated 600,000 patients annually in the United States) and mortality (50,000–200,000 deaths)^1^. Currently, it is the third most common cause of cardiovascular death, following coronary artery disease and stroke world widely^2^. The rapid diagnosis and treatment is beneficial to patients with PE. The diagnosis of PE is mainly based on clinical signs and symptoms, D-dimer measurements and contrast-enhanced pulmonary CT angiography^3–5^. Contrast-enhanced pulmonary CT angiography allows the pulmonary arteries to be viewed to at least the sub-segmental level and is currently considered the preferred imaging method for diagnosing PE^5,6^. Therefore, CTPA is widely used in patients suspected of PE, which can lead to the overuse of CTPA and unnecessary radiation exposure to patients.

Many strategies are being developed to limit the radiation dose, including minimizing tube current, reducing tube voltage, the automatic tube current technique, and iterative reconstruction algorithms^7–9^. Low tube voltage technique achieves increased attenuation in the vessel by exploiting the k-edge of iodine, and therefore improves the enhancement in vessels. Thus, a CTPA protocol with low tube voltage and low iodine may improve or maintain image quality^10,11^. ASIR ﻿(the adaptive statistical iterative reconstruction technique, ASIR) is a way to improve image noise using “a reconstructed image through modeling fluctuations in projection data due to photon statistics and electronic system noise”^12^, as a useful technique to improve SNR (signal to noise ratio, SNR) and CNR(contrast to noise ratio, CNR) whilst reducing contrast and radiation dose. Recent studies^10,13,14^ have shown that CT angiography protocol with low tube voltage, low iodine and ASIR results in an effective reduction in the radiation dose and iodine content of contrast agent whilst maintaining image quality.

To acquire high iodine concentrations in pulmonary arteries as well as good image quality, the current CTPA protocols mainly adopt high concentrations and large doses of iodine contrast agent^15,16^. It was previously shown^17^ that the incidence of contrast-induced nephropathy increased as the contrast agent’s dose increased. And the viscosity of the contrast agent increased along with the concentration of contrast agent. Therefore, it is very important to use low concentration of contrast agents and use doses of contrast agent that are as low as possible without affecting CTA imaging. In recent decades, low concentration of contrast agent is advanced into the clinic, such as 240 mg I/ml^18^ and 270 mg I/ml^17^. Low concentration of contrast agent is proved with a lower viscosity and gets an acceleration of contrast agent distribution within blood. Furthermore, the peak injection pressure of contrast agent with low concentration is lower with fewer complications (such as extravasation) compared to contrast agent with high concentration^19^.

The objective of this study was to compare the image quality, radiation dose, positive rate of detecting PE and the location of PE between CTPA protocols using low tube voltage (80 KVp) and low-iodine-concentration contrast agent (270 mg I/ml) in patients suspected of having PE with those of a conventional CTPA protocol (120 kVp, 350 mg I/ml).

## Materials and Methods

### Patients

Present study was approved by the Ethics Committee of Tongji Hospital Affiliated to Tongji Medical College of Huazhong University of Science and Technology. All participants provided written informed consent and all methods were performed in accordance with the relevant guidelines and regulations. Between November 2013 and June 2016, outpatients and inpatients that were clinically suspected of PE were enrolled consecutively in this study and required CTPA examinations. Inclusion criteria: 1, Patients suspected of PE based on clinical signs and symptoms(acute chest pain and dyspnea), abnormal plasmatic D-dimer level and/or deep vein thrombosis in the lower extremity; 2, Patients aged >18 years old; 3 patients with a body mass index (BMI) <30 kg/m^2^; 4, patients with a glomerular filtration rate >60 ml/min/1.73 m^2^. Exclusion criteria: 1, Patients with history of allergy to iodinated contrast agent; 2, patients with comorbidities such as severe pneumonia and atelectasis. A total of 382 patients were randomly divided into two groups. One hundred ninety-two patients (group A, double low group) were examined with the 80 kVp/40 ml 270 mg I/ml (iodixanol, GE Healthcare, Carrigtohill, Co:Cork, Ireland) protocol, and 190 patients (group B, contrast group) were examined with the 120 kVp/40 ml 350 mg I/ml (Ioversol, tyco Healthcare, Pointe-Claire, Quebec Canada) protocol. The demographic data are shown in Table 1. There were no statistically significant differences in age, gender or BMI between two groups.

**Table 1:** The patients’ demographic data of two groups (A, double low group; B, contrast group).

Group	Patients	Gender		Age (years)	BMI (kg/m^2^)
		Male	Female		
A	192	99	93	53.3 ± 15.2	23.5 ± 2.8
B	190	101	89	54.5 ± 13.6	23.6 ± 2.3
P value		0.760		0.413	0.779

### CTPA examination

All CTPA examinations were performed using a commercially available 64-MDCT scanner (Discovery CT750 HD, GE healthcare). Patients were examined in a supine position, and both arms were extended above the head. All CTPA data were acquired in the craniocaudal direction from the lung apex to the costophrenic angles during a single breath-hold (inspiration). The bolus-tracking technique was used in the main pulmonary artery with a trigger attenuation threshold of 50 HU. Total 40 ml of contrast agent was injected. First, 12 ml contrast agent and 18 ml saline was injected simultaneously at a flow rate of 4 ml/s using a dual-head power injector in order to compensate for a delay time of 7 s when the table moved from the bolus-tracking level to the lung apex. Then, the rest 28 ml contrast agent injected followed by a constant saline flush of 30 ml at a flow rate of 5 ml/s. The automatic tube current technique was employed with a noise index of 18 HU. The data reconstruction of group A (80 kVp/40 ml 270 mg I/ml) used 40% ASIR and 60% FBP(filt﻿er back projection, FBP), however, the data of group B (120 kVp/40 ml 350 mg I/ml) was reconstructed only using FBP. The technical parameters of CTPA for two groups were shown in Table 2.

**Table 2:** The technical parameters of CTPA for two groups (A, double low group; B, contrast group).

	Group A	Group B
Dose of contrast agent	40 ml	40 ml
Iodine concentration of Contrast agent	270 mg I/ml	350 mg I/ml
Tube voltage	80 kVp	120 kVp
Tube current	auto	auto
Noise index	18 HU	18 HU
Pitch	1.375	1.375
Slice thickness	0.625 mm	0.625 mm
Tube rotation time	0.5 s	0.5 s
convolution kernel	Standard	Standard
Reconstruction	40% ASIR and 60% FBP	100% FBP

### Image analysis and radiation exposure dose

The CTPA data were transferred to an external workstation (Advantage Windows 4.5, GE Healthcare). The quantitative analysis was performed by a radiologist with more than 5 years of experience. The CT attenuation in the nine arteries was measured using a ROI that was slightly less than the selected artery: the pulmonary trunk, left pulmonary artery, right pulmonary artery, both upper lobe arteries, both lower lobe arteries, right middle lobe artery and left lingual artery. The reader may adjust the window level to avoid the pulmonary emboli. Then, the average value was calculated as the mean CT attenuation. The average standard deviation in the three regions (left, middle and right) with a ROI area of 20 mm^2^ in front of the chest at the right pulmonary artery slice was measured as the background noise. The CT value of the bilateral paravertebral muscle was measured and the mean value was calculated as the background CT attenuation. The signal-to-noise ratio (SNR) was calculated as the mean CT attenuation/the background noise. The contrast-to-noise ratio (CNR) was calculated as follows: (mean CT attenuation-background C﻿T attenuation)/background noise.

The subjective image quality analysis and the diagnosis of PE were performed by two radiologists with more than 4 years of experience who were blinded to the clinical features and the CTPA protocols. The displayed pulmonary branch level and the severity of iodine contrast agent beam hardening artifacts in the superior vena cava were recorded. The pulmonary arteries were graded as follows: the first pulmonary arterial branch (left and right pulmonary arteries), the second pulmonary branch (lobe arteries), the third pulmonary branch (segmental arteries), the fourth pulmonary branch (sub-segmental arteries), the fifth pulmonary branch and the sixth pulmonary branch. For assessment of the displayed level of pulmonary branches, a coronal MIP-reconstruction  image standard with 40mm-slice thickness, 800 Hu-window width and 80 Hu-window center was used. The CTPA images needed to display at least the fourth pulmonary branch to satisfy the requirement of diagnosis. The severity of the iodine contrast agent beam hardening artifacts in the superior vena cava was graded as follows: mild, gentle artifacts that did not affect the measurement; moderate, a few artifacts affected the measurement in the right pulmonary artery; and severe, severe artifacts affected the measurement in the right pulmonary artery and the upper lobe artery. The two radiologists discussed and agreed on diagnosis of PE and the location of pulmonary emboli was recorded. The readers may adjust the window level for diagnosis of PE.

The dose length product (DLP) was available from the CTPA protocols recorded automatically. The effective dose (ED) was calculated using DLP, ED = k * DLP.

The K value adopted was 0.017, as shown by the European Commission (CEC)^20^.

### Data analysis

Statistical analysis was performed using the SPSS 19.0 software package. All quantitative data were described as the means ± standard deviation. The independent samples t-test was used to compare age, BMI, mean CT value, noise, SNR, CNR and ED between the groups. Gender, the severity of iodine contrast agent beam hardening artifacts in the superior vena cava, the displayed pulmonary branch level, the positive rate for detecting PE and the location of pulmonary emboli were analyzed between the groups using the chi-square test. A p value < 0.05 was considered to represent statistical significance. The inter-observer agreement for the subjective image was assessed using Kappa analysis.

## Results

**Figure 1:**
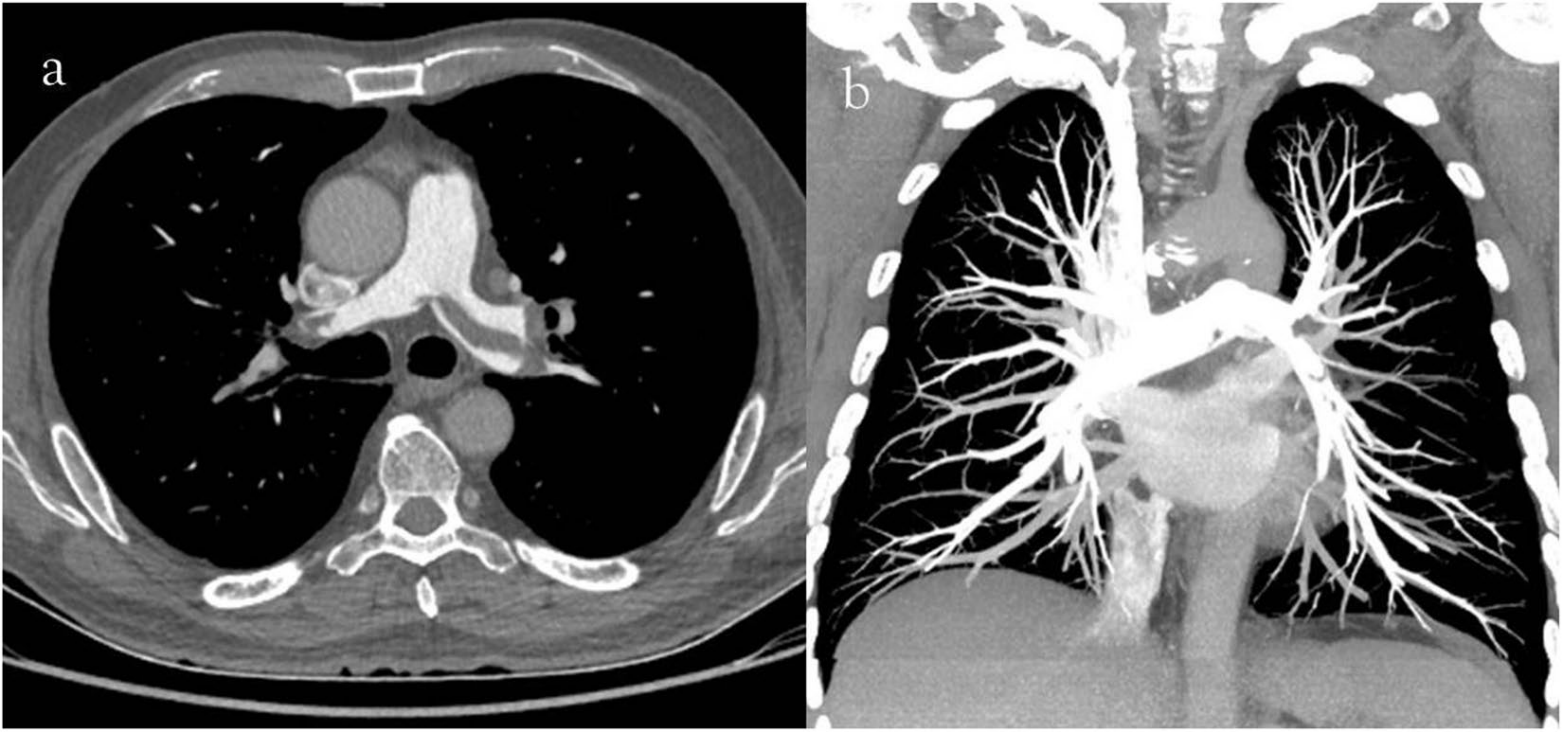
(**a**) 64-year-old man with pulmonary embolism. The CTPA performed with 80 kVp/40 ml 270 mg I/ml. Mild iodine contrast agent beam hardening artifacts in the superior vena cava (**a**, axial thin slice image) were observed, and 5–6 pulmonary arterial branches (**b**, coronal MIP image with 800 Hu-window width and 80 Hu-window center) were displayed.

**Figure 2:**
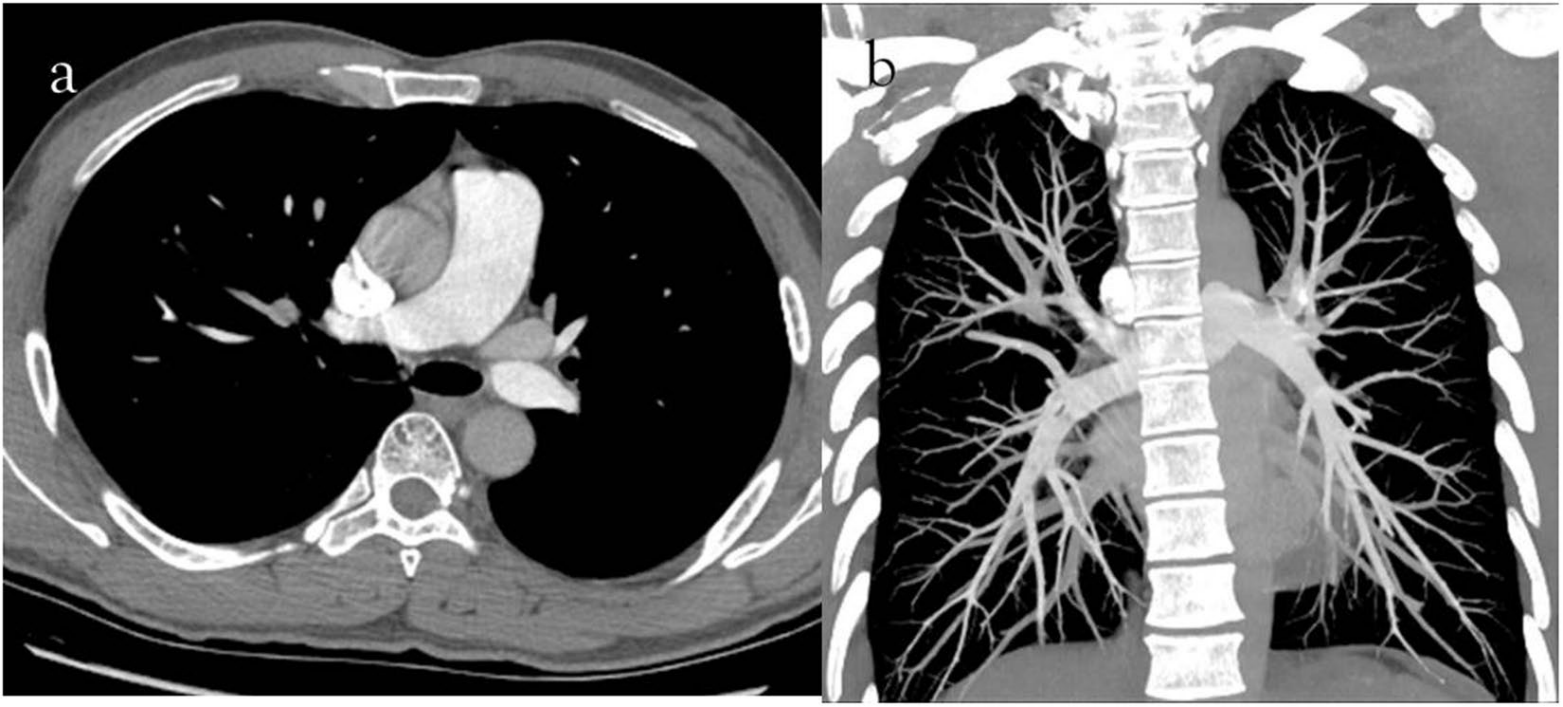
(**a**) 40-year-old man with pulmonary embolism. The CTPA performed with 120 kVp/40 ml 350 mg I/ml. Severe iodine contrast agent beam hardening artifacts in the superior vena cava (**a**, axial thin slice image) were observed, and 5–6 pulmonary arterial branches (**b**, coronal MIP image with 800 Hu-window width and 80 Hu-window center) were displayed.

**Figure 3:**
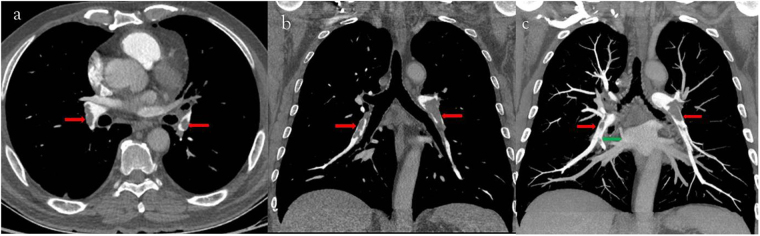
(**a**) 64-year-old man with pulmonary embolism. The CTPA performed with 80 kVp/40 ml 270 mg I/ml. Filling defects were seen in bilateral lower lobe arteries (**c**, axial thin slice image; **d**, coronal thin slice image; **e**, coronal MIP image; red arrow) and in a segmental artery (**e**, green arrow).

**Figure 4:**
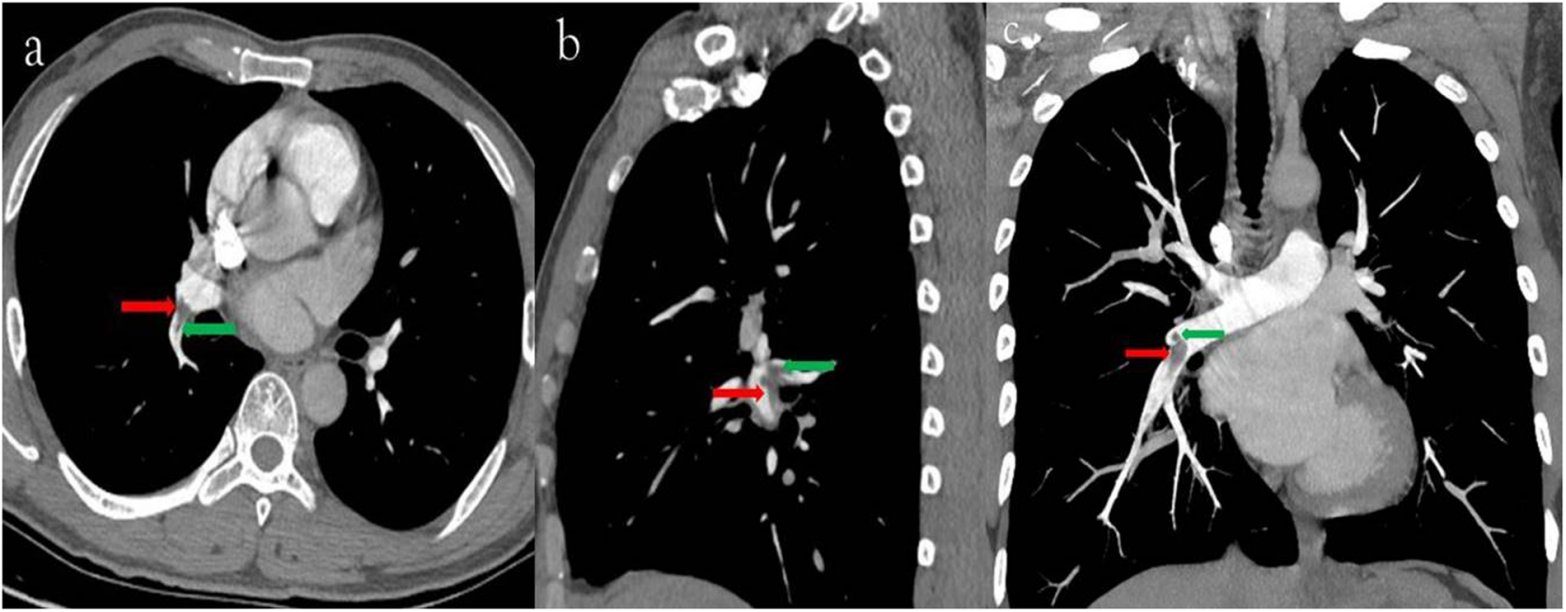
(**a**) 40-year-old man with pulmonary embolism. The CTPA performed with 120 kVp/40 ml 350 mg I/ml. Filling defects were seen in the right inferior lobe artery (**c**, axial thin slice image; d, sagital thin slice image; **e**, coronal MIP image; red arrow) and in the dorsal segmental artery (**c**, **d** and **e**, green arrow).

Both the mean CT value in the pulmonary arteries and the background noise were significantly higher in group A than in group B, whereas the SNR and CNR were lower in group A than group B (Table 3). There was no significant difference in the displayed pulmonary arterial branch level and severity of iodine contrast agent beam hardening artifacts in the superior vena cava between the groups, with good inter-observer agreement (Tables 4 and 5, Figs 1 and 2). There was no statistically significant difference between the groups in terms of the positive rate for detecting PE and the location of pulmonary emboli (Tables 3 and 6, Figs 3 and 4). The ED in group A was significantly lower than that in group B (Table 3). The radiation exposure in group A was reduced by nearly 63.6% compared to that in group B, and the iodine content administered in group A was decreased by 22.9% relative to group B.

**Table 3:** Image quantitative analysis, radiation dose and the positive rate for detecting PE in the groups (A, double low group; B, contrast group).

	Mean CT value	Noise	SNR	CNR	ED	Diagnosis	
						Positive	Negative
Group A	419.5 ± 121.9	18.0 ± 3.9	24.5 ± 9.4	20.3 ± 8.9	2.0 ± 0.9	55	137
Group B	342.3 ± 98.6	12.3 ± 2.4	29.1 ± 10.8	23.6 ± 10.2	5.5 ± 0.8	64	126
P value	0.000	0.000	0.000	0.001	0.000	0.320

**Table 4:** Results of the displayed pulmonary arterial branches level and severity of the iodine contrast agent beam hardening artifacts in the superior vena cava for two groups (A, double low group; B, contrast group).

	Artifacts						Level					
	Reader 1			Reader 2			Reader 1			Reader 2		
	Mild	Moderate	Severe	Mild	Moderate	Severe	4	5	6	4	5	6
Group A	67	71	54	75	63	54	48	94	50	38	91	63
Group B	86	63	41	88	55	47	54	104	32	49	90	51
P value	0.099			0.358			0.089			0.265

**Table 5:** The inter-observer agreement for the subjective image.

Artifacts				Level			
Reader 1	Reader2			Reader1	Reader2		
	Mild	Moderate	Severe		4	5	6
Mild	146	7	0	4	83	19	0
Moderate	17	94	23	5	4	159	35
Severe	0	17	78	6	0	3	79
Kappa value	0.744				0.746

**Table 6:** The location of pulmonary emboli in two groups (A, double low group; B, contrast group).

Pulmonary arterial branch level	1	2	3	4	5
Group A	9	34	50	33	6
Group B	9	36	59	44	7
P value	1.000	0.857	0.332	0.185	0.985

## Discussion

The study demonstrated that CTPA protocol with 80 kVp and 40 ml 270 mg I/ml acquired equally subjective image quality and a positive rate for diagnosing PE, though the quantitative image quality was reduced compared to the conventional CTPA protocol (120 kVp, 40 ml 350 mg I/ml). It also achieved a 63.6% decrease in radiation dose and a 22.9% reduction in iodine content of contrast agent.

With respect to patient health, reducing the radiation exposure during CT examinations is one of the most important goals of CT scientists and clinical operators. A decrease in radiation dose can be accomplished by reducing the tube voltage and the tube current. In theory, the radiation dose is proportional to the square of tube voltage^21^. Therefore, the low tube voltage scanning technique is an effective method for decreasing the radiation dose and has become a hotspot of research over the last decade. Several studies^6,21,22^ have demonstrated that the CTPA protocol with 80 kVp achieved a 50–70% reduction in the radiation dose compared to the 120 kVp CTPA protocol when the other parameters remained unchanged. The automatic tube current modulation technique can adjust the tube current according the individual size and anatomy to acquire stable image quality and an 18% decrease of radiation exposure dose compared to fixed tube current technique^8^. In our study, we employed low tube voltage combined with the automatic tube current modulation technique to minimize radiation exposure dose and achieved a nearly 63.6% reduction in the radiation exposure dose, as expected.

An advantage of the low tube voltage technique is that the attenuation in the vessel increases because the energy level moves closer to the K-edge of iodine contrast agent, thus potentially decreasing the dose of contrast agent^10^. In the present study, although the iodine content administered to patients in the 80 kVp group was lower, the mean CT value in the pulmonary arterial branches was still higher than that observed in the 120 kVp group. The results of our study were in line with those of other researchers^10,21–23^.

A disadvantage of the low tube voltage technique is increased image noise due to the decreased X-ray energy level. An article by Zamboni^22^ demonstrated that the noise in the segmental pulmonary arteries or above was higher in the 80-kv protocol than in the 120-kv protocol. In our study, the mean standard deviation of the air in front of the chest was chosen as the background noise to avoid the influence of other tissues, referencing the article by Szucs-Farkas^24^. The CT scanner used in our study was equipped with ASIR, which has a satisfactory effect on noise elimination. A number of researchers^9,25–27^ have found that ASIR was a useful tool to decrease the radiation dose while maintaining the image quality; furthermore, the noise was decreasing along with the percentage of ASIR increasing. But 30–50% ASIR for chest^27–29^ and 40–60% ASIR for coronary CTA^30^ was recommended, based on the subjective image quality assessment. So we used a 40%ASIR for our low voltage protocol. The results of our study showed that the background noise in the low tube voltage group was still higher than that in the conventional tube voltage group, though ASIR was employed. One possible reason is that decreased background noise by 40% ASIR + 60% FBP cannot compensate for the increased background noise by reducing tube voltage from 120 kVp to 80 kVp. A higher percentage of ASIR may be need for CTPA protocol with low tube voltage.

Recently, large doses of a high-iodine-concentration contrast agent, such as 400 mg I/ml and 370 mg I/ml, were administered to achieve high enhancement in the vessels^1,22^. Considering the harm that iodine contrast agent inflicts on the kidney and cardiovascular system, contrast agents with low iodine concentrations are clinically well accepted. Iodixanol (270 mg I/ml) has a low viscosity and is easy to mix with blood after injected into a blood vessel. The pulmonary circulation is faster, and pulmonary artery reinforcement would reach a peak at 7–13 s after the contrast agent is injected. If the scan time is calculated exactly and the optimum dose of contrast agent is administered, we can obtain high-quality images with reduced superior vena cava iodine contrast agent beam hardening artifacts. In our study, only 40 ml of contrast agent was used, which was less than the doses reported by other studies^15,31^. However, the results were not quite satisfactory. Though the CTPA protocol using 40 ml of contrast agent could display the pulmonary arteries to at least the sub-segmental level, the iodine contrast agent beam hardening artifacts in vena cava were equal between the groups, and severe iodine contrast agent beam hardening artifacts in the vena cava were observed in many cases. This means that there is probably room to further minimize the dose of contrast agent. Some literatures demonstrated that it was feasible for contrast agent reduced to 20–30 ml for CTPA protocols without compromising diagnostic image quality^23,32^. But different injection and scanning protocols were needed, such as bolus testing technique and different trigger position (the superior vena cava). The advantage of our study is that a larger number of samples and lower concentration of contrast agent was used.

In recent decade, the dual-energy CT scanner was introduced to clinic, which could obtain conventional CT images, virtual monenergistic images and accurate material decomposition images (such as iodine-based material decomposition images)^33^. Previous studies have confirmed that the optical monenergisteic images (range of 65–70 keV)^34^ could improve image quality and diagnostic confidence for detection of PE and the iodine maps correlated to SPECT/CT^35^ and MR perfusion^36^ could detect the peripheral pulmonary emboli missing in CTPA images. That means it is feasible to minimize the dose of contrast agent to less than 40 ml with maintaining even improving image quality and the diagnostic performance for PE using dual energy CT scanner. It may be one of the research orientations in the future.

In our study, both the mean CT value and the background noise were increasing in low tube voltage group, but the SNR and CNR were decreasing compared to regular tube voltage group. This result indicates that the reduced quantitative image quality using low tube voltage and low concentration contrast agent was not in line with expectations. One probable reason is that reducing tube voltage from 120 kVp to 80 kVp generated much background noise. A 22.9% reduction of iodine concentration may be another reason. Though the quantitative image quality of CTPA protocol using low tube voltage and low iodine concentration of contrast agent was decreased, the displayed pulmonary arterial level and the positive rate for detecting PE were equal as conventional CTPA protocol with high iodine concentration of contrast agent. That means CTPA protocol with low tube voltage and low iodine concentration of contrast agent is satisfied to the clinical application.

### Limitations

There are some limitations to our study. First, individual doses of contrast agent were not used. A dose of 40 ml contrast agent for all patients may have affected image quality. Second, pulmonary arterial branches of grades 5–6 were not displayed in a minority of cases. Thus, the pulmonary emboli in such pulmonary arteries may be missed, and false negative results will appear in these cases, although the positive rate of detecting PE is not the focus of this article. Additionally, multiple percentages of ASIR were not used in this study to find optical percentage of ASIR for CTPA protocol with low voltage tube and low dose of contrast agent. Finally, we limited the use of CTPA protocol with low tube voltage and low iodine concentration to patients with BMI <30 kg/m^2^.

## Conclusion

Compared to a conventional CTPA protocol, the CTPA protocol with low tube voltage and low-iodine-concentration contrast agent achieved at least equally subjective image quality and the positive rate of diagnosing PE, with an approximately 63.6% decrease in radiation dose and a 22.9% reduction in the iodine content of the contrast agent.
