# Author Correction: Use of pulmonary CT angiography with low tube voltage and low-iodine-concentration contrast agent to diagnose pulmonary embolism

**DOI:** 10.1038/s41598-018-22948-9

**Published:** 2018-03-12

**Authors:** Xuemei Hu, Liya Ma, Jinhua Zhang, Zhen Li, Yaqi Shen, Daoyu Hu

**Affiliations:** Department of Radiology, Tongji Hospital Affiliated to Tongji Medical College of Huazhong University of Science and Technology, Wuhan, China

Correction to: *Scientific Reports* 10.1038/s41598-017-13077-w, published online 16 October 2017

This Article contains an error in the legend of Figure 3.

“(a) 64-year-old man with pulmonary embolism. The CTPA performed with 80 kVp/40 ml 270 mg I/ml. Filling defects were seen in bilateral lower lobe arteries (c, axial thin slice image; d, coronal thin slice image; e, coronal MIP image; red arrow) and in a segmental artery (e, green arrow).”

should read:

“(a) 64-year-old man with pulmonary embolism. The CTPA performed with 80 kVp/40 ml 270 mg I/ml. Filling defects were seen in bilateral lower lobe arteries (red arrows) and in a segmental artery (green arrow). (a, axial thin slice image; b, coronal thin slice image; c, coronal MIP image).”

This Article also contains an error in the legend of Figure 4.

“(a) 40-year-old man with pulmonary embolism. The CTPA performed with 120 kVp/40 ml 350 mg I/ml. Filling defects were seen in the right inferior lobe artery (c, axial thin slice image; d, sagital thin slice image; e, coronal MIP image; red arrow) and in the dorsal segmental artery (c, d and e, green arrow).”

should read:

“(a) 40-year-old man with pulmonary embolism. The CTPA performed with 120 kVp/40 ml 350 mg I/ml. Filling defects were seen in the right inferior lobe artery (red arrow) and in the dorsal segmental artery(green arrow) (a, axial thin slice image; b, sagittal thin slice image; c, coronal MIP image).”

